# Epidemiological characteristics and temporal-spatial analysis of overseas imported dengue fever cases in outbreak provinces of China, 2005–2019

**DOI:** 10.1186/s40249-022-00937-5

**Published:** 2022-01-24

**Authors:** Xinchang Lun, Yiguan Wang, Chunchun Zhao, Haixia Wu, Caiying Zhu, Delong Ma, Mingfang Xu, Jun Wang, Qiyong Liu, Lei Xu, Fengxia Meng

**Affiliations:** 1 State Key Laboratory of Infectious Diseases Prevention and Control, National Institute for Communicable Disease Control and Prevention, Chinese Center for Disease Control and Prevention, Beijing, 102206 People’s Republic of China; 2 ChangSha Center for Disease Control and Prevention, Changsha, 410004 Hunan People’s Republic of China; 3 School of Public Health and Health Management, Shandong First Medical University and Shandong Academy of Medical Sciences, Jinan, 250117 Shandong People’s Republic of China; 4 Vanke School of Public Health, Tsinghua University, Beijing, 100084 People’s Republic of China

**Keywords:** Imported case, Dengue fever, China, Epidemiology, Temporal-spatial distribution

## Abstract

**Background:**

Overseas imported dengue fever is an important factor in local outbreaks of this disease in the mainland of China. To better prevent and control such local outbreaks, the epidemiological characteristics and temporal-spatial distribution of overseas imported dengue fever cases in provincial-level administrative divisions (PLADs) where dengue fever is outbreak in the mainland of China were explored.

**Methods:**

Using the Chinese National Notifiable Infectious Disease Reporting Information System (CNNDS), we identified overseas imported dengue fever cases in dengue fever outbreak areas in the mainland of China from 2005 to 2019 to draw the epidemic curve and population characteristic distribution of overseas imported cases in each PLAD. Based on spatial autocorrelation analysis of ArcGIS 10.5 and temporal-spatial scanning analysis of SaTScan 9.5, we analyzed the temporal-spatial distribution of overseas imported dengue fever in dengue fever outbreak areas in the mainland of China.

**Results:**

A total of 11,407 imported cases, mainly from Southeast Asia, were recorded from 2005 to 2019 in these 13 PLADs. Of which 62.1% were imported into Yunnan and Guangdong Provinces. Among the imported cases, there were more males than females, mainly from the 21–50 age group. The hot spots were concentrated in parts of Yunnan, Guangdong and Fujian Provinces. We found the cluster of infected areas were expanding northward.

**Conclusions:**

Based on the analysis of overseas imported dengue cases in 13 PLADs of the mainland of China from 2005 to 2019, we obtained the epidemiological characteristics and spatial distribution of imported dengue cases. Border controls need to pay attention to key population sectors, such as 21–50 years old men and education of key populations on dengue prevention. There is a need to improve the awareness of the prevention and control of imported cases in border areas. At the same time, northern regions cannot relax their vigilance.

**Graphical Abstract:**

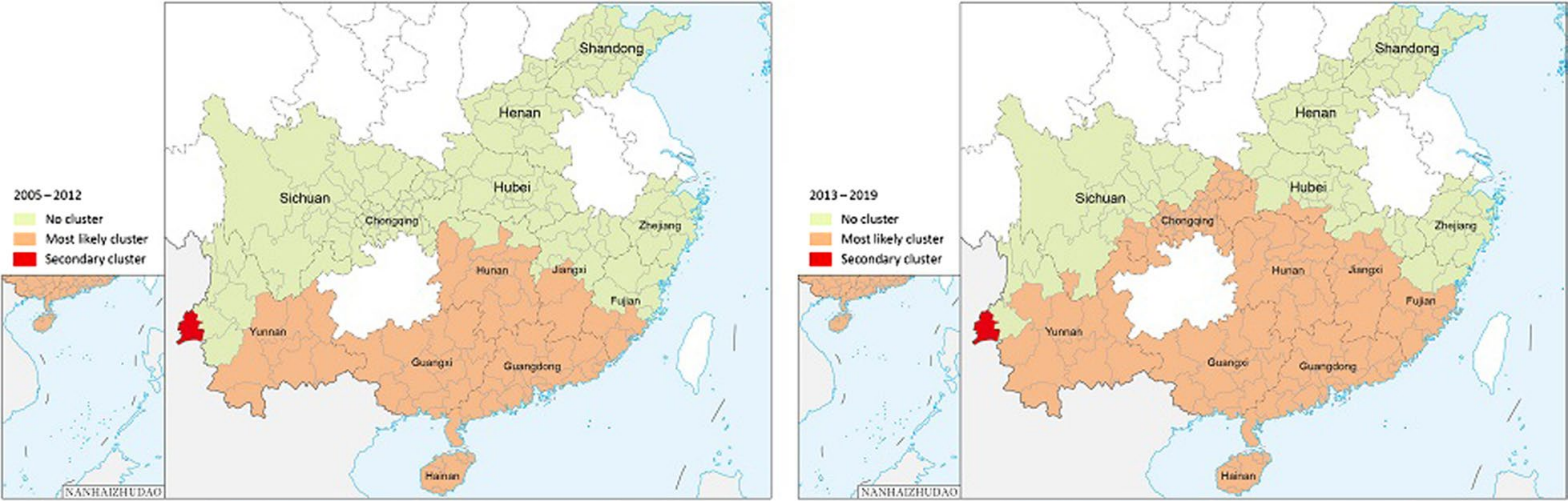

**Supplementary Information:**

The online version contains supplementary material available at 10.1186/s40249-022-00937-5.

## Background

Dengue fever is a mosquito-borne disease that is caused by the dengue virus and vectored by *Aedes albopictus* and *Aedes aegypti* [1–3]. Dengue virus can be divided into four serotypes (DENV-1, DENV-2, DENV-3 and DENV-4) [4, 5]. As early as 1780, Benjamin Rush described a dengue fever epidemic in Philadelphia [6]. Before 1970, only nine countries recorded dengue epidemics [7]. However, the prevalence of dengue fever expanded dramatically to 128 countries by 2012 [8]. It was estimated that there were 390 million dengue infections worldwide in 2010 with 70% of cases in Asia [9]. The clinical manifestations of dengue fever are diverse, including fever, nausea, vomiting, skin rash and other symptoms. In severe cases, shock and death can occur [10, 11]. Dengue fever creates major economic and social burdens on affected countries and poses a great threat to public health [12].

The first outbreak of dengue fever in the mainland of China since 1949 occurred in Guangdong Province in 1978, and the province has had the most frequent outbreaks of dengue fever in the mainland of China [13–15]. In 2014, the number of dengue fever cases in Guangdong reached its peak with the number of reported cases exceeding 45,000 [16]. Furthermore, the geographic range of indigenous dengue fever in the mainland of China is gradually expanding northward [^17^]. As of 2019, there had been local outbreaks of dengue fever in 13 provincial-level administrative divisions (PLADs), and the northernmost line of the dengue fever outbreak had arrived in Jining city, Shandong Province in 2017 [18].

Imported cases of dengue is an important factor affecting local outbreaks of dengue fever in the mainland of China [19, 20]. As of 2019, overseas imported cases had been reported in all PLADs in the mainland of China except the Tibet Autonomous Region [21]. More importantly, the number of imported cases increased each year from 2005 to 2016. Most of the cases were imported from neighboring Asian countries [22]. These imported cases often lead to local outbreaks of dengue fever [23, 24]. However, few studies have focused on the epidemiological characteristics and temporal-spatial distribution of imported dengue fever cases in China.

Here we summarize the overseas imported dengue fever cases in 13 PLADs where there were local outbreaks of dengue fever from 2005 to 2019. We analyze the epidemiological characteristics and the temporal-spatial distribution of the cases in these PLADs. Based on the analysis we propose different prevention measures for geographical areas with different dengue outbreak characteristics. This study provides a basis for the reasonable allocation of medical resources and the effective prevention and control of dengue fever.

## Materials and methods

### Study area

As of 2019, there had been local outbreaks of dengue fever recorded in 13 PLADs in the mainland of China, namely, Hainan, Yunnan, Guangxi, Guangdong, Fujian, Hunan, Jiangxi, Sichuan, Zhejiang, Hubei, Henan and Shandong, and Chongqing City (see Additional file 1).

### Data sources

#### Cases data

The dengue case data we used in this study were retrieved from the Chinese National Notifiable Infectious Disease Reporting Information System (CNNDS). We selected clinically diagnosed and confirmed cases of dengue fever from 1 January 2005 to 13 December 2019 based on the date of onset of disease symptoms. We used the 13 PLADs with local outbreaks of dengue fever as research sites to explore the population epidemic characteristics of overseas imported dengue cases, at the same time, the temporal-spatial distribution of overseas imported cases was discussed at the municipal level. The sources of dengue fever cases were distinguished as indigenous dengue cases, overseas imported dengue cases, domestic imported dengue cases and cases of unknown origin. Indigenous dengue cases are cases that had not left the local city where they lived 14 days before the onset of illness. Overseas imported dengue cases are defined as those that had been to a dengue-endemic country or region within 14 days before the onset. Domestic imported dengue cases are cases that had left their current city of residence and went to another city in the country within 14 days before the onset of the disease, and cases of unknown origin are cases whose origin cannot be identified. In this study, we used overseas imported dengue cases as the research content. Overseas imported cases included both Chinese and foreign nationalities.

#### Geographical coordinate data

We imported the vector maps of 13 PLADs into ArcGIS 10.5 (ESRI, Redlands, CA, USA) and obtained their latitude and longitude coordinates by transforming the x and y planar coordinates of each city. These data are used for temporal-spatial scanning analysis.

#### Demographic data

From the statistical yearbooks, we obtained the annual permanent population data or household registration population data of each city from 2005 to 2019. These data are used for temporal-spatial scanning analysis.

### Statistical analysis

WPS Office 2021 software (Kingsoft, Beijing, China) was used to sort the epidemiological characteristics of overseas imported dengue cases, including import source country or region, import region distribution, time characteristics and demographic distribution characteristics such as gender distribution, age distribution, and occupation distribution, in 13 PLADs from 2005 to 2019.

ArcGIS 10.5 software was used to perform spatial autocorrelation analysis of the overseas imported dengue cases. Spatial autocorrelation analysis [25], which includes global autocorrelation and local autocorrelation, can be used to analyze the spatial correlation of a variable. In this study, we used global Moran's *I* [26, 27] and Getis-Ord General G analysis [28, 29] to estimate the global autocorrelation. The value range of Moran's *I* index is between −1 and 1. When Moran's *I* index is closer to 1, the positive spatial correlation is stronger, that is, there is a spatial aggregation relationship between regions; when Moran's *I* index is closer to −1, the negative spatial correlation is stronger, which means that the attributes between the regions are opposite; and when Moran's *I* index is closer to 0, it means that the spatial distribution of the variables in the research area follows a random distribution pattern. However, the index cannot determine the specific aggregation mode. The Getis-Ord General G index can indicate the degree and quantity of high and low values and is used to analyze the correlation of a variable between adjacent areas. The null hypothesis indicates that there is no space clustering for the research variable in the adjacent study area. When the calculated *P* value is statistically significant, we can judge it by observing the Getis-Ord general G value and *Z* score. When the Getis-Ord general G value is higher than the expected value and the *Z* score is positive, it indicates that the high value is clustered, that is, there is a hot spot; and when the Getis-Ord general G value is lower than the expected value and the *Z* score is negative, it indicates that the low values have a tendency to gather, that is, there is a cold spot.

Global Moran's *I* and Getis-Ord General G analysis can use the spatial statistics tools module in the ArcGIS software. The above two analyses are in the analyzing patterns of the spatial statistics tools module.

The local autocorrelation was analyzed using the Anselin local Moran's *I* [30, 31] and Getis-Ord Gi* [32]. The Anselin local Moran's *I* index, as a local index of spatial correlation, represents the spatial correlation between a research area and its neighboring areas for the research variables, including low-low, low-high, high-low, and high-high, and not significant. The low-low and high-high types indicate spatial aggregation, that is, cold spots and hot spots, respectively. These suggest that an area with a small number of imported cases of dengue fever is surrounded by a neighboring area with a small number of imported cases and an area with a large number of imported cases of dengue fever is surrounded by a neighboring area with a large number of imported cases, respectively. The low-high and high-low types indicate spatial outliers, which respectively indicate that an area with a small number of imported cases of dengue fever is surrounded by adjacent areas with a large number of imported cases and an area with a large number of imported cases of dengue fever is surrounded by an adjacent area with a small number of imported cases. Through Getis-Ord Gi* analysis, the specific spatial clustering positions of high and low values can be observed, and the specific distribution of cold spots and hot spots can be identified.

The Anselin Local Moran's *I* and Getis-Ord Gi* can use the spatial statistics tools module in the ArcGIS software. The above two analyses are in the mapping clusters of the spatial statistics tools module.

We used the SaTScan 9.5 software (Martin Kulldorff, Boston, MA, USA) to perform the temporal-spatial scanning analysis of overseas imported cases. The SaTScan 9.5 software detects the accumulation of a certain disease in time and space by scanning the studied areas using a cylindrical scan window. The log-likelihood ratio (*LLR*) can be used to identify the locations of the most likely clusters and secondary clusters and other clustering regions. Under Poisson's assumption, the log-likelihood ratio of the scanning window is:

$$LLR=({\frac{\mathrm{c}}{\mathrm{E}\left[\mathrm{c}\right]})}^{\mathrm{c}}({\frac{\mathrm{C}-\mathrm{c}}{\mathrm{C}-\mathrm{E}\left[\mathrm{c}\right]})}^{\left(\mathrm{C}-\mathrm{c}\right)}I()$$

where C represents the total number of cases, c represents the number of observed cases in the window, and E(c) represents the expected number of cases adjusted by covariates in the window under the condition of the null hypothesis. Here, the null hypothesis is that for window scanning, the aggregation trend inside the window is the same as that outside of the window. C−E[c] represents the expected number of cases outside the window, and I() represents the indicator function. The *LLR* is related to the scan rate of the cluster. When the cluster is scanned only at a high rate and the number of cases in the window is higher than the expected number of cases under the null hypothesis, I() = 1; otherwise, I() = 0.

The Poisson model was used to analyze the spatial aggregation area. The circular window is selected as the scanning window, and the largest scanning area is 50% of the total population. The *P* value was determined by the combination of the Standard Monte Carlo method, Sequential Monte Carlo method and Gumbel Approximation. The test level ɑ = 0.05, and the number of Monte Carlo iterations was set to 999. The temporal-spatial scanning analysis was visualized using the ArcGIS 10.5 software.

## Results

### Overview of overseas imported dengue fever cases in the 13 PLADs

#### Sources of the imported cases

From 2005 to 2019, a total of 11,407 imported cases of dengue fever were reported in the 13 PLADs in China. Among these cases, 11,105 were imported from the Asian region, accounting for 97.3%. Specifically, the cases from Southeast Asia accounted for 91.9%. In addition, Africa, Oceania, South America and North America were sources of imported cases, accounting for 1.6%, 0.5%, 0.3% and 0.1%, respectively. The sources of the remaining 0.2% of imported cases were unknown.

Among the overseas imported cases in each PLAD, the proportions of Chinese and foreign nationals are slightly different. Foreign cases in Yunnan Province were the highest, accounting for 44.0%; but accounted for less than 10% in the other PLADs (Table 1).

**Table 1:** Proportion of Chinese nationality and foreign nationality cases among overseas imported cases, 2005–2019

PLAD	Chinese nationality	Proportion (%)	Foreign nationality	Proportion (%)	Total
Yunnan	2,608	56.0	2,052	44.0	4,660
Guangdong	2,235	92.3	187	7.7	2,422
Fujian	1,018	92.2	86	7.8	1,104
Zhejiang	941	94.9	51	5.1	992
Sichuan	434	96.9	14	3.1	448
Hunan	434	98.6	6	1.4	440
Henan	261	100.0	0	0.0	261
Hubei	252	99.6	1	0.4	253
Chongqing	240	100.0	0	0.0	240
Jiangxi	200	99.5	1	0.5	201
Shandong	147	94.2	9	5.8	156
Guangxi	139	93.9	9	6.1	148
Hainan	74	90.2	8	9.8	82

*PLAD* provincial-level administrative division.

#### Distribution and epidemic trend of imported cases

**Fig. 1:**
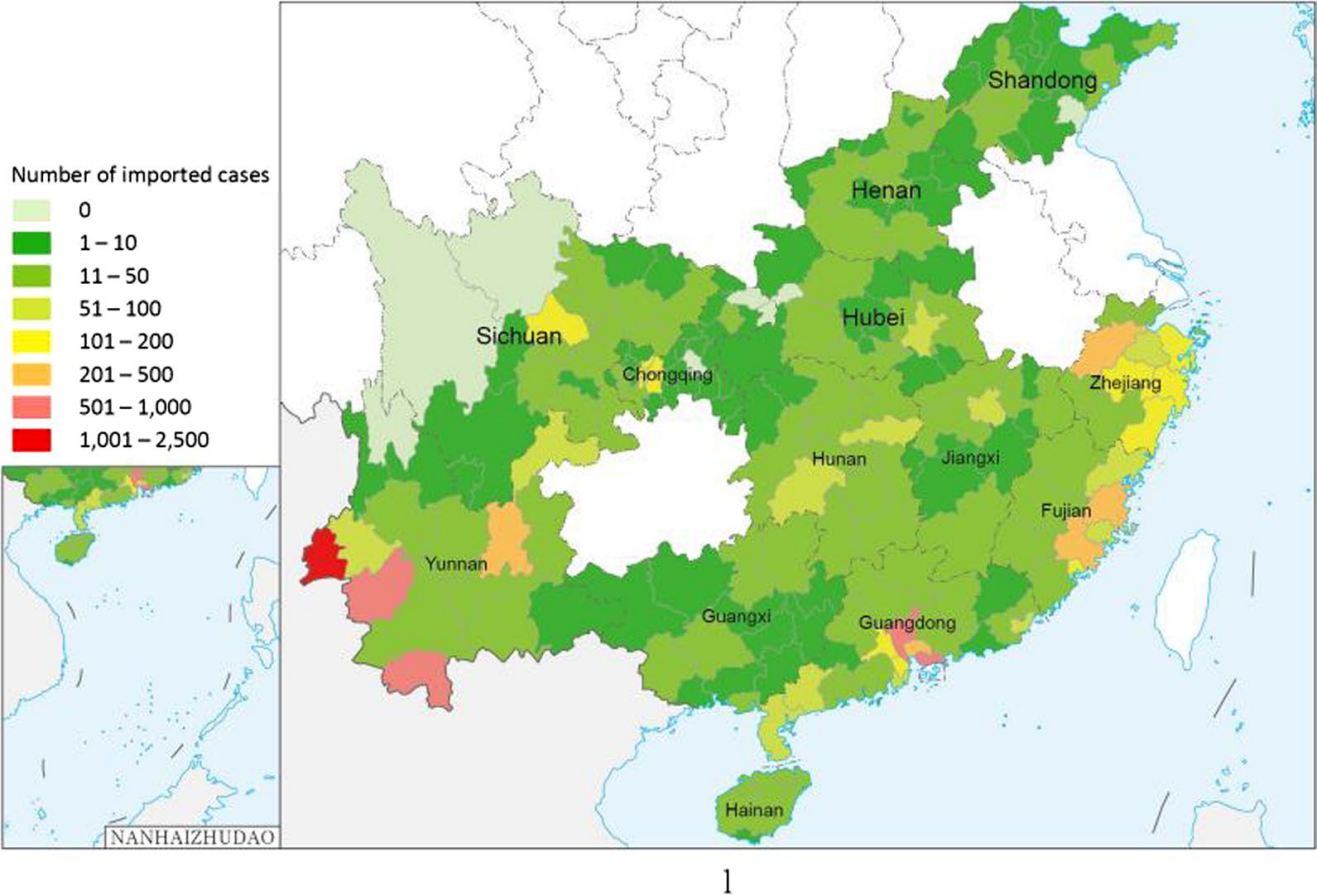
Distribution of overseas imported dengue fever cases, 2005–2019

All the municipalities in the 13 studied PLADs, with a few exceptions for the northwestern part of Yunnan Province, the northwestern part of Sichuan Province, the Shennongjia forest region of Hubei Province and Rizhao city of Shandong Province and a few areas in Chongqing, had imported dengue fever cases from 2005 to 2019 (Fig. 1). The municipal area in western Yunnan Province bordering Myanmar had the largest number of overseas imported cases, reaching 2,485 cases, accounting for 21.8% of the total overseas imported cases in all 13 PLADs. In general, overseas imported cases are more likely to be observed in the border areas of Yunnan Province, the southeast coastal areas and the capital areas with developed economies and convenient transportation (Fig. 1).

**Fig. 2:**
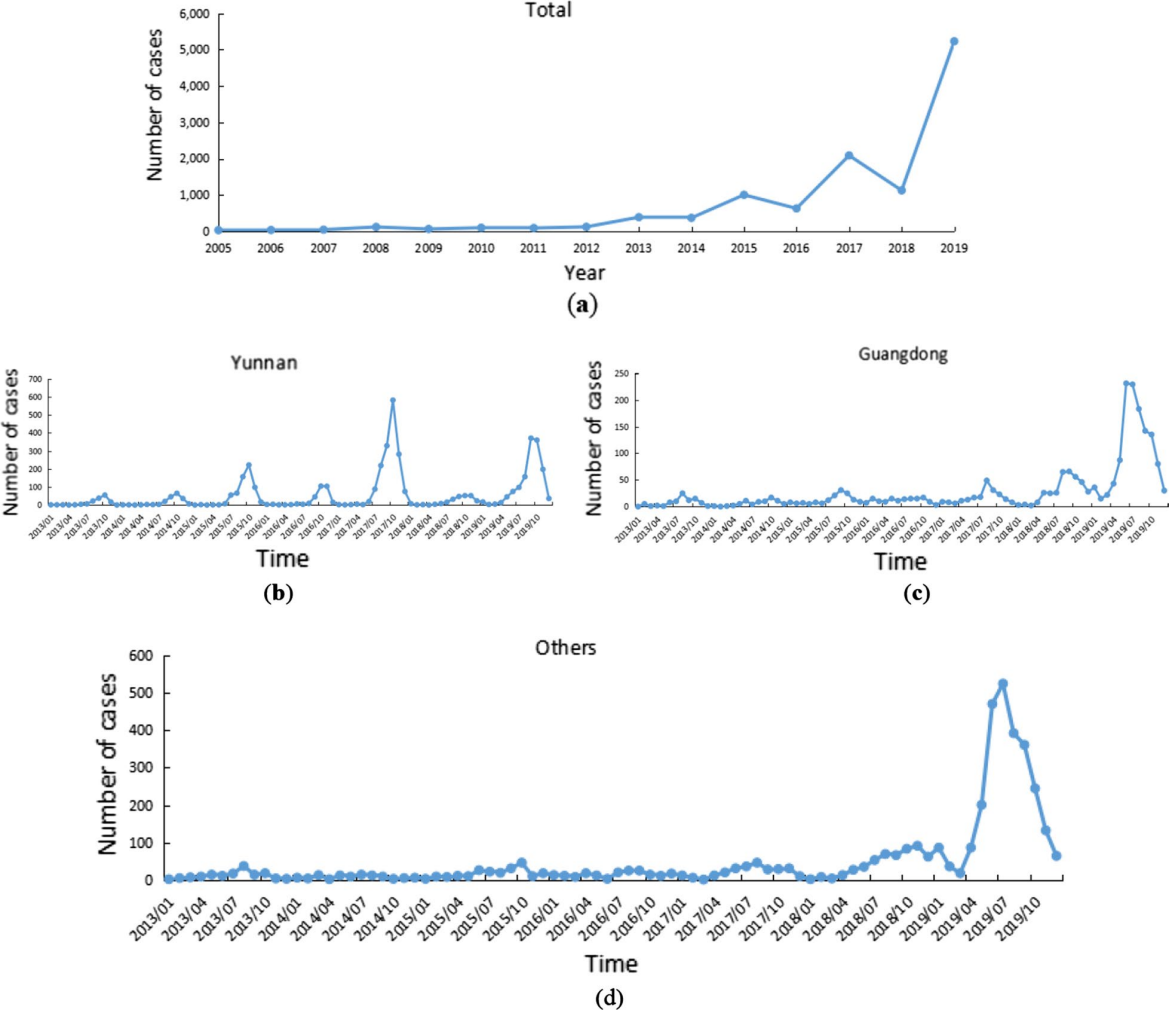
The trend of the number of overseas imported dengue fever cases, 2005–2019 for total (**a**), 2013–2019 for Yunnan (**b**), Guangdong (**c**) and all other Provincial-level administrative divisions combined (**d**)

Overseas imported dengue fever cases in most of the provinces began to increase from 2013 and accounted for 94.9% of the total cases (Table 2 and Fig. 2), so here we focus on the prevalence of imported dengue fever cases from 2013 to 2019. During this period, Yunnan Province and Guangdong Province reported a large proportion of imported cases, accounting for 62.2% of the total. The prevalence curve of these cases from 2013 to 2019, in provinces with more imported cases, showed a clear seasonal trend with most from July to November (Fig. 2).

**Table 2:** The proportion of overseas imported dengue fever cases for each PLAD overall (2005–2019) and broken into two periods, 2005–2012 and 2013–2019

PLAD	2005–2012		2013–2019		2005–2019	
	No. of overseas imported dengue fever cases	Proportion (%)	No. of overseas imported dengue fever cases	Proportion (%)	No. of overseas imported dengue fever cases	Proportion (%)
Yunnan	177	30.5	4,483	41.4	4,660	40.9
Guangdong	173	29.8	2,249	20.8	2,422	21.2
Fujian	95	16.4	1,009	9.3	1,104	9.7
Zhejiang	40	6.9	952	8.8	992	8.7
Sichuan	15	2.6	433	4.0	448	3.9
Hunan	28	4.8	412	3.8	440	3.9
Henan	4	0.7	257	2.4	261	2.3
Hubei	13	2.2	240	2.2	253	2.2
Chongqing	4	0.7	236	2.2	240	2.1
Jiangxi	9	1.5	192	1.8	201	1.8
Shandong	7	1.2	149	1.4	156	1.3
Guangxi	10	1.7	138	1.2	148	1.3
Hainan	6	1.0	76	0.7	82	0.7
Total	581	100.0	10,826	100.0	11,407	100.0
	5.1%		94.9%		100.0%	

*PLAD* provincial-level administrative division.

### Population distribution characteristics of overseas imported dengue fever cases in the 13 PLADs

#### Gender characteristics

**Fig. 3:**
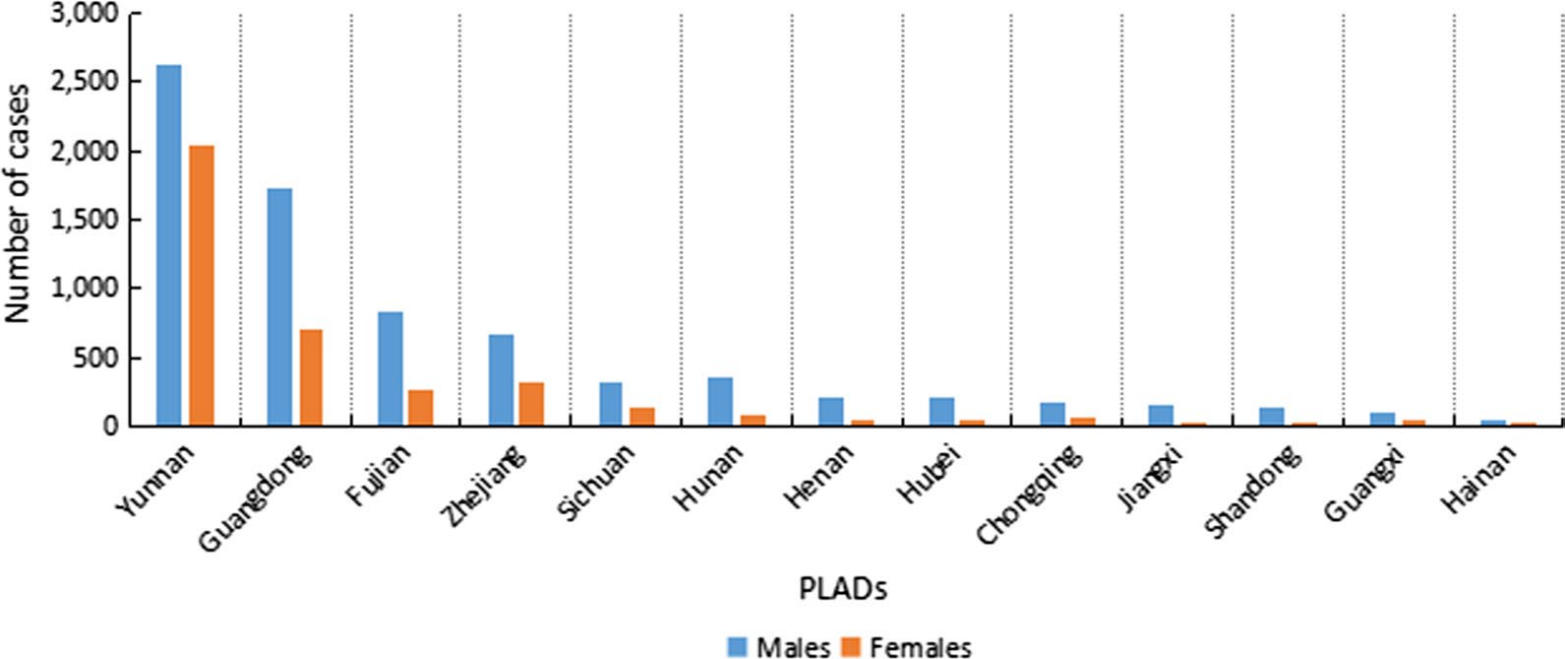
Gender distribution of overseas imported dengue fever cases, 2005–2019. *PLADs* Provincial-level administrative divisions

The number of males imported cases was higher than females from 2005 to 2019, with a male-to-female ratio of 2.0:1 (Fig. 3). The ratios in Yunnan Province and Hainan Province were relatively low, which were below the overall male-to-female ratio in the 13 PLADs, at 1.3:1 and 1.8:1, respectively. The highest ratio was observed in Shandong Province, at approximately 6:1.

#### Age characteristics

**Fig. 4:**
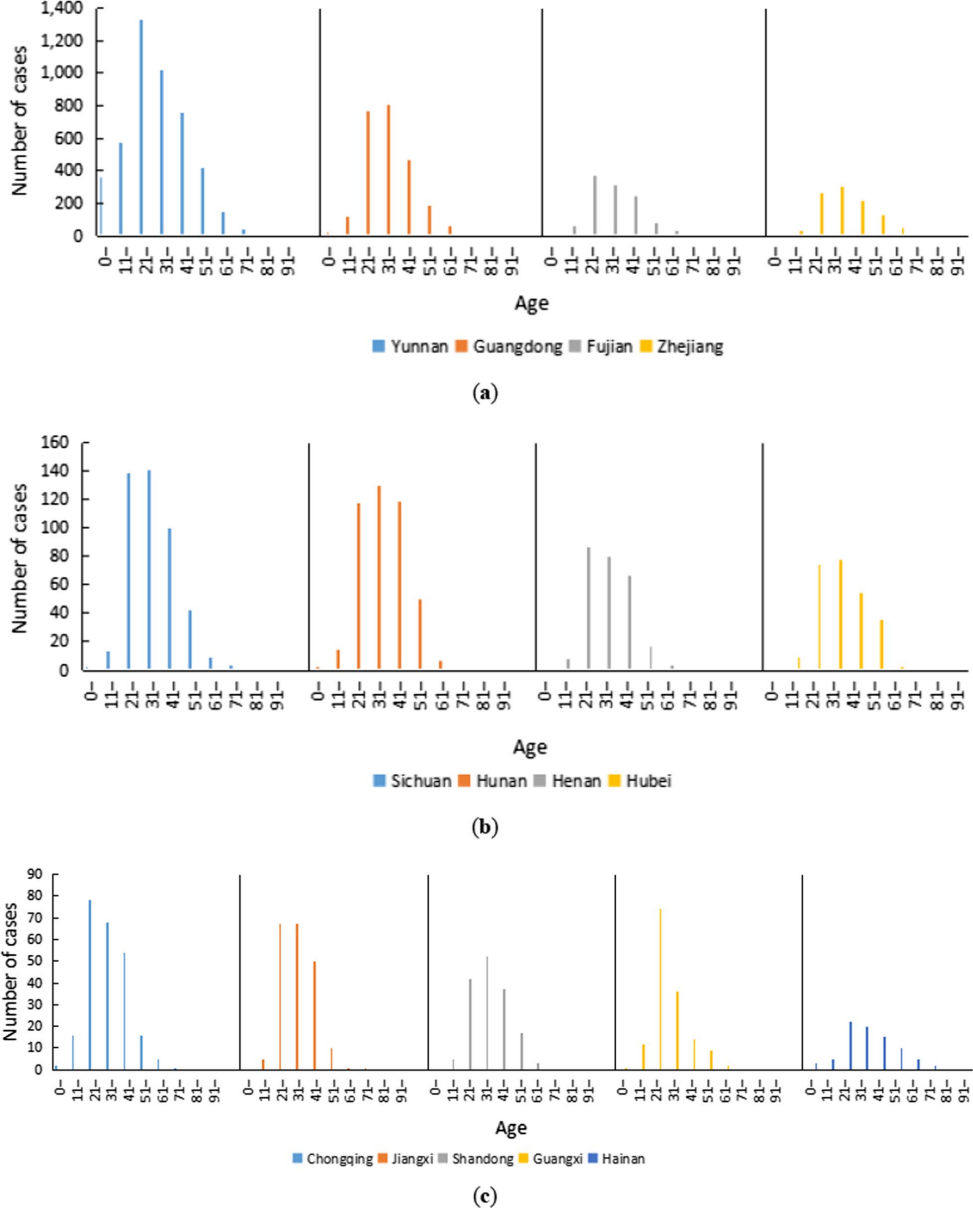
Age characteristics of overseas imported dengue fever cases, 2005–2019

The age distribution of the imported dengue fever cases in the 13 PLADs exhibited similar patterns (Fig. 4). The young and middle-aged people aged 21–50 accounted for a large proportion (76.6%) of the imported cases. In Yunnan, Fujian, Henan, Chongqing, Guangxi and Hainan, the numbers of imported cases were the largest in the 21–30 age group and the second-largest imported cases in the 31–40 age group. In Jiangxi Province, there were the same number of people in these two age groups, and both were the most. The imported cases in other PLADs were more concentrated in the 31–40-year-old age group, followed by the 21–30-year-old age group.

#### Occupational characteristics

**Fig. 5:**
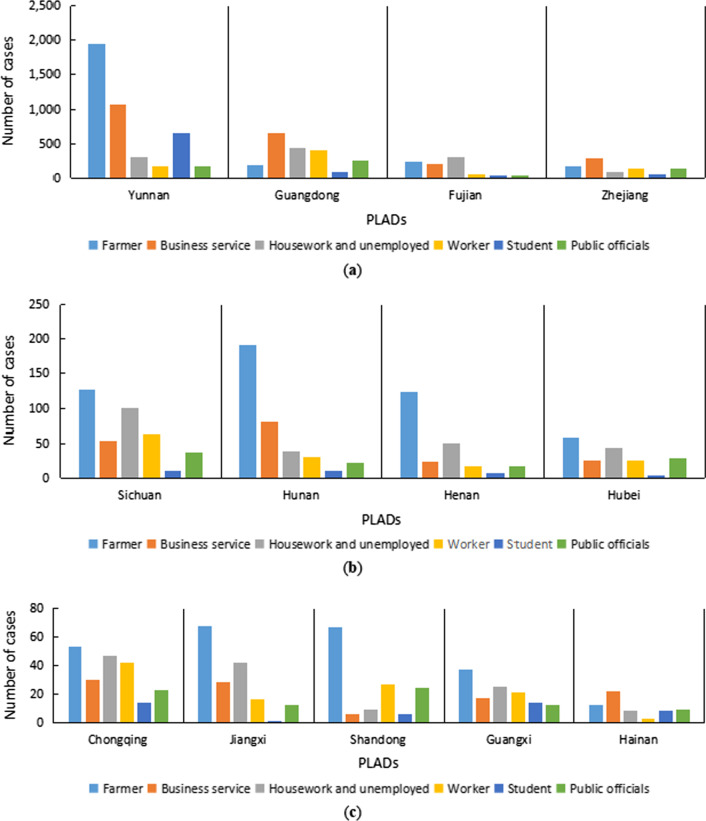
Occupational characteristics of overseas imported dengue fever cases, 2005–2019

Farmers, business services, housework or unemployed, workers, students and public officials accounted for 87.7% of the total number of overseas imported cases. Those above occupations showed slightly different proportions in different provinces. Farmers accounted for the highest proportion of imported cases in Yunnan, Sichuan, Hunan, Henan, Hubei, Chongqing, Jiangxi, Shandong and Guangxi. In contrast, the proportion of commercial services was highest in Guangdong, Zhejiang and Hainan. Different from other PLADs, the occupation with the highest proportion of overseas imported cases in Fujian was housework and unemployed (Fig. 5) (Additional file 2).

### Temporal-spatial distribution characteristics of overseas imported dengue fever cases in the 13 PLADs

#### Global autocorrelation statistics

The global Moran's *I* index as 0.11 and the *Z* score as 3.6 (*P* < 0.01) for all imported cases from 2005 to 2012. The results suggested that in the 13 PLADs with local outbreaks of dengue fever, the numbers of imported cases from abroad were clustered in the spatial distribution at the municipal level. The Getis-Ord General G value was estimated as 0.000001, and the *Z* score was estimated as 2.5 (*P* < 0.05), suggesting a tendency for high clusters of overseas imported dengue fever cases from 2005 to 2012. That is, municipal areas with more overseas imported cases and more overseas imported cases were aggregated in the spatial distribution.

From 2013 to 2019, the global Moran's *I* index was 0.08, and the *Z* score was 3.6 (*P* < 0.01). The results showed that in the 13 PLADs with local outbreaks of dengue fever, the numbers of imported cases from abroad were clustered in the spatial distribution at the municipal level. The Getis-Ord General G value was 0.000001, and the *Z* score was 1.8 (*P* < 0.1), suggesting a tendency for high clusters of dengue fever cases imported from 2013 to 2019. That is, municipal areas with more overseas imported cases and more overseas imported cases were aggregated in the spatial distribution.

#### Local autocorrelation analysis

**Fig. 6:**
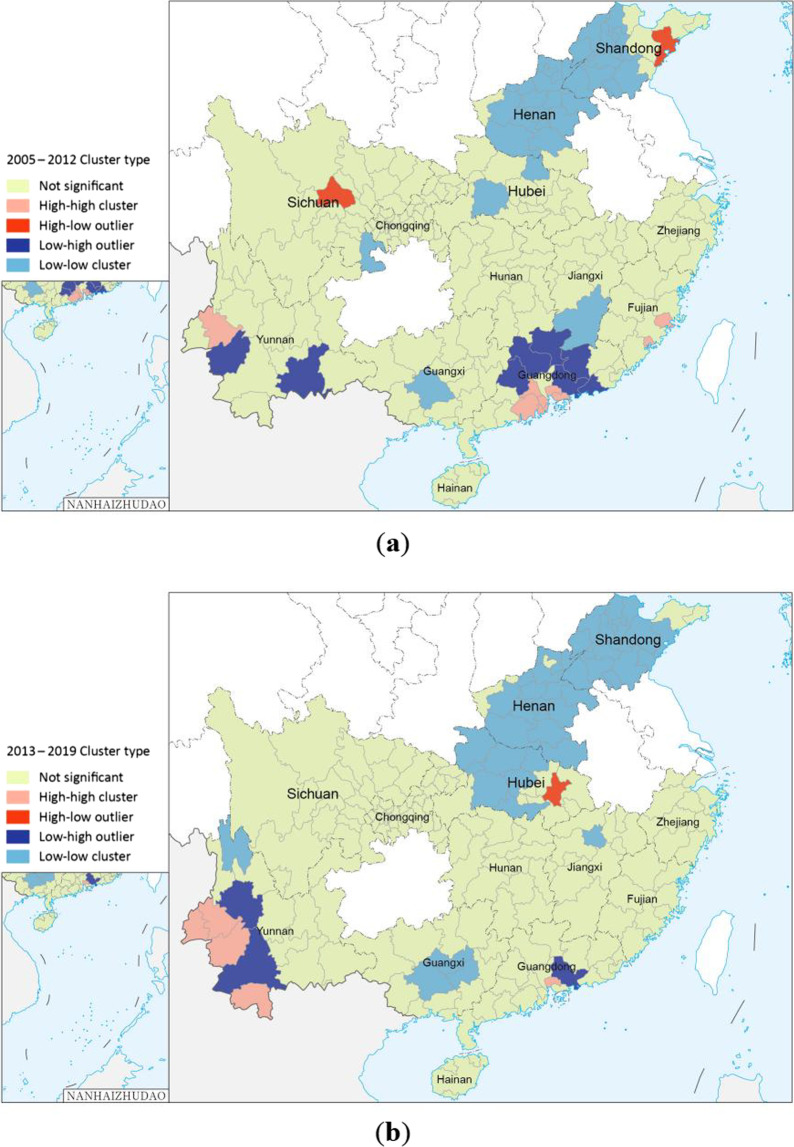

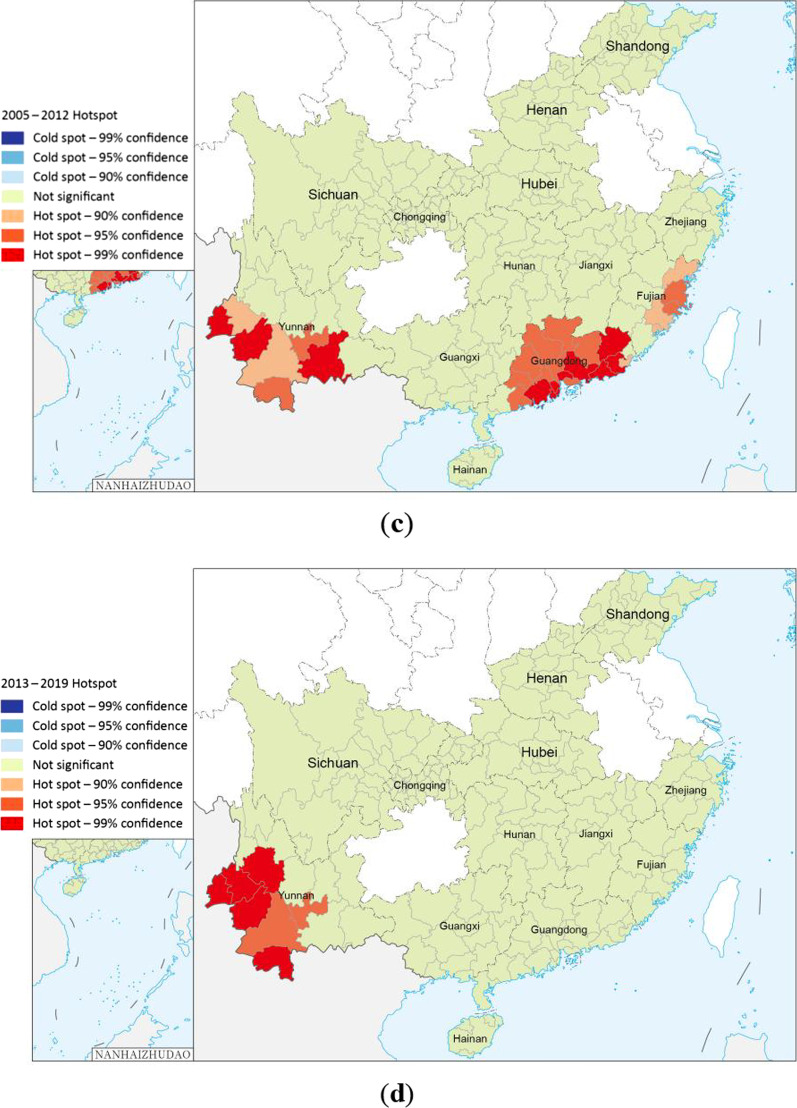
Local spatial autocorrelation of overseas imported dengue fever cases, 2005–2019. (**a** is the result of the Anselin local Moran's *I* analysis in 2005–2012, **b** is the result of the Anselin local Moran's I analysis in 2013–2019, **c** is the result of the Getis-Ord Gi* analysis in 2005–2012, and **d** is the result of the Getis-Ord Gi* analysis in 2013–2019.)

The Anselin local Moran's *I* analysis of the imported cases from 2005 to 2012 revealed high-high clusters at the border area of Yunnan, southeastern Guangdong, and two coastal areas in Fujian. In contrast, low-low clusters were detected in most areas of Henan and Shandong and in one area or two areas in Guangxi, Jiangxi, Sichuan, and Hubei, respectively. Sichuan and Shandong had one region each that was a high-low cluster. Two border areas in Yunnan and the other 6 cities in the northern part of Guangdong were identified as low-high clusters (Fig. 6a).

From 2013 to 2019, the number of PLADs with local spatial autocorrelation areas decreased. Four border areas in Yunnan and a region in southeastern Guangdong were high-high clusters. The central part of Guangxi, the central part of Hubei, Henan and Shandong were mostly low-low clusters. The capital of Hubei was a high-low cluster. Two border areas in Yunnan and a city in eastern Guangdong were low-high clusters (Fig. 6.b).

Getis-Ord Gi* analysis showed that the imported cases from 2005 to 2012 had formed hot spots in some areas in Yunnan, Guangdong, and Fujian, including the border areas of Yunnan, most parts of Guangdong and the coastal areas of Fujian (Fig. 6c). However, hot spots of the imported cases during 2013–2019 were only identified in Yunnan (Fig. 6d).

#### Temporal-spatial distribution characteristics of overseas dengue fever cases

**Fig. 7:**
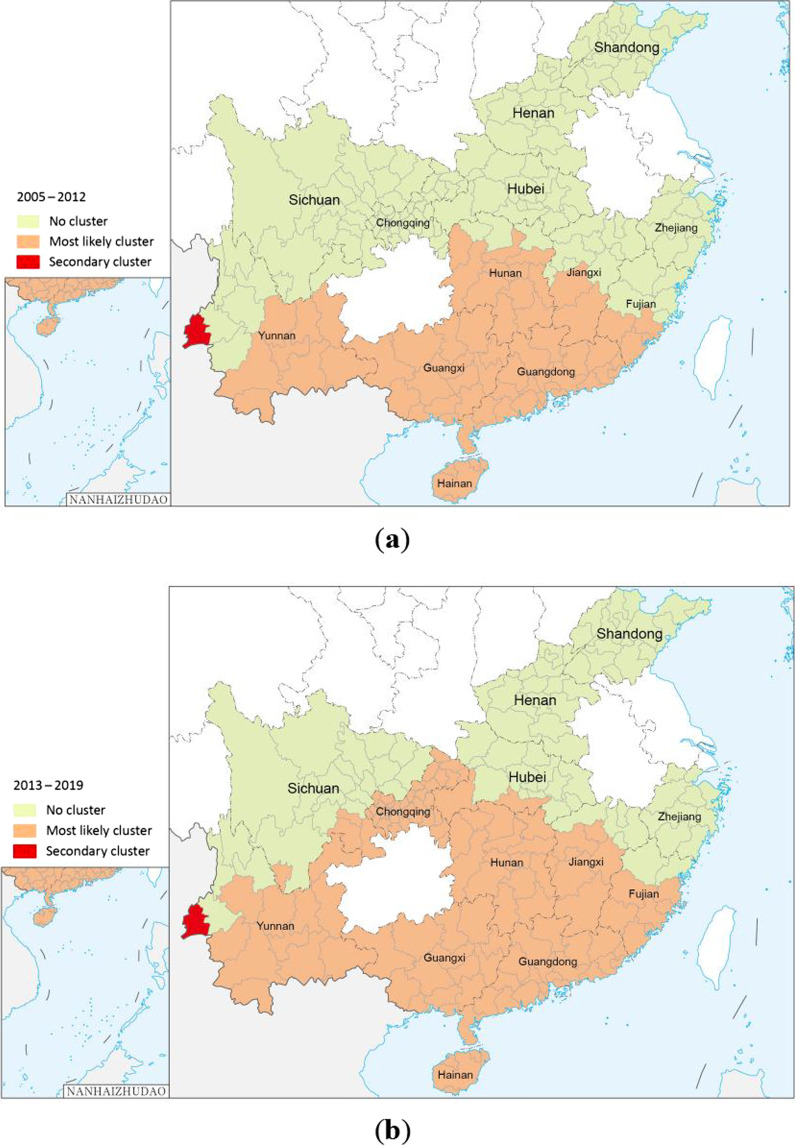
Distribution of spatial clusters of overseas imported dengue fever cases, 2005–2019. **a** is the result of the temporal-spatial scan analysis from 2005 to 2012, and **b** is the result of the temporal-spatial scan analysis from 2013 to 2019

Using SaTScan 9.5, we used the maximum spatial cluster size of 50% of the at-risk population to scan the clustering areas in the two time periods of 2005–2012 and 2013–2019. The results showed that the Dehong Dai and Jingpo Autonomous Prefectures in Yunnan were the most likely clusters for both time periods (*P* < 0.01). The regional scope of the secondary cluster was significantly larger from 2013 to 2019. During 2005–2012, secondary clusters were identified in Hainan, eastern Yunnan, Guangxi, Guangdong, central and southern Hunan, southern Jiangxi, and southern Fujian (*P* < 0.01). During 2013–2019, the secondary cluster expanded westward and northward, reaching the areas around Chongqing (*P* < 0.01) (Fig. 7, Table 3).

**Table 3:** Temporal-spatial scan results of overseas imported dengue fever, 2005–2019

Period	Cluster	Aggregation time	No. of observed cases	No. of expected cases	Relative risk	*LLR*	*P* value
2005–2012	I	2008/8/1–2008/11/30	46	0.04	1,321.4	282.7	< 0.01
	II	2010/7/1–2012/12/31	223	66.4	4.8	140.4	< 0.01
2013–2019	I	2014/8/1–2017/12/31	1,986	8.6	282.1	9,019.8	< 0.01
	II	2019/5/1–2019/11/30	3,266	463.8	9.7	3,991.1	< 0.01

*LLR*: The log-likelihood ratio can be used to identify the locations of the most likely clusters and secondary clusters and other clustering regions.

## Discussion

Dengue fever in the mainland of China is an imported vector-borne disease and has not yet formed epidemics [33], So, imported cases is an important factor affecting local outbreaks of dengue fever in the mainland of China. A better understanding of the epidemiological characteristics and temporal-spatial distribution of imported cases will be of great significance for the prevention and control of dengue fever, and provide a theoretical basis for the rational allocation of medical resources. In this study, we analyzed over eleven thousand overseas imported dengue fever cases in 13 PLADs in China from 2005 to 2019, including the locations of these cases, the onset dates, the gender and age distributions and the occupations. We also investigated the geographic autocorrelation of these cases and identified the hot spots and spatial clusters.

Most overseas imported cases were located in the border areas of Yunnan; the coastal areas of Zhejiang, Fujian, and Guangdong; provincial capitals of Chongqing city and some other provinces. The reasons for these locations being mostly affected by import cases may vary considerably. Yunnan which lies in southwestern China, is one of the provinces with the longest border. It shares a border that is approximately 4,060 km with Myanmar, Laos, and Vietnam. It is also a key region connecting Southeast Asia and South Asia. Given the prevalence of dengue fever in Southeast Asia and South Asia [34, 35] and the frequent cross-border migrants, this region has been a high-risk area for imported dengue fever. In contrast, the coastal areas of Zhejiang, Fujian, and Guangdong and the provincial capitals of Chongqing city and some other provinces, despite the absence of borders with neighboring countries, have relatively developed economies and high trade activities with large population flows. These factors increase the likelihood of importing cases of dengue fever cases. Studies [36, 37] have shown that a series of infectious diseases, such as dengue fever, have been imported into China amid the rapid development of globalization, the increase of migrants caused by tourism, and the exchange of a large number of laborers and products. The number of imported dengue fever cases in China was closely related to the number of tourists from Southeast Asia and the number of overseas workers [37].

In a province such as Yunnan that has a long border with neighboring countries, cases imported via ground transportation may account for a large proportion of the total cases. Ground transportation includes road transportation by foot, car, bus, truck, etc., and by railway. For trade and family visits among border residents, frequent exchanges make it difficult to control. In contrast, in coastal provinces such as Guangdong without terrestrial international borders, more cases may be imported by air. Air transportation provides a better grasp of the passengers' departure location, destination and other information; and keep the conditions of passenger flow, time and space relatively fixed. Given these differences, prevention strategies should also be different. For the former case, attention should be given to the prevalence of dengue fever in China's neighboring countries; strengthening the publicity and education of border residents on dengue fever-related knowledge, including dengue fever prevention, symptoms, timely medical treatment and other related knowledge; and strengthening the management of border residents' mobility. For the latter case, we should pay close attention to countries with large amounts of air transportation with China, monitor the temperatures of passengers, place brochures on dengue fever prevention and control knowledge in airport waiting rooms, and publicize and educate passengers who go to dengue epidemic areas and return from dengue epidemic areas.

In the 13 PLADs studied the number of overseas imported dengue fever cases reached a peak in 2019, which may be closely related to the intensity of the dengue fever epidemic in neighboring countries. According to WHO statistics, the year with the highest number of reported dengue fever cases in the world was 2019. All WHO regions were affected [7]. This year was also the year with the largest number of overseas imported cases in these 13 PLADs. Among the areas affected by dengue fever, the dengue fever epidemic in Southeast Asia, which is the largest source of imported cases, is very serious. For example, Myanmar is a country with a high burden of dengue fever in the Asia-Pacific region. During 2011–2015, there were 89,832 dengue-related admissions. In 2017 and 2018, there were 31,288 and 23,273 dengue fever patients, respectively [38]. A large number of cases were imported from Cambodia [39]. This may be related to the fact that after Chinese businessmen establish new factories in Cambodia, they summoned a large number of multinational migrant workers to work in Cambodia [40]. For provinces with a large number of imported cases, such as Yunnan and Guangdong, most of the imported cases were reported between July and November, which overlapped with the local outbreak of dengue fever in the mainland of China [21, 41–43]. Studies have shown that rainfall, temperature and humidity are important climatic factors affecting the density of mosquitoes [44, 45], and the climatic conditions in this period are conducive to the breeding of mosquitoes. Therefore, the measures to prevent and control overseas imported dengue fever should be intensified between July and November.

In terms of gender, the number of imported cases from 2005 to 2019 was greater for men than women. This may be related to the difference in the social division of labor between men and women. Men more frequently move across borders, whether for business purposes or as migrant workers, which greatly exposes them to dengue fever. In terms of age, most of the imported cases were between 21 and 50 years old. Given that people in this age group are more likely to be laborers, they have a greater likelihood of contracting dengue fever.

In terms of occupation, the difference in the distributions in the 13 PLADs reflects a distinct economic status and overall occupational difference of residents in each PLAD. For example, the imported cases in the developed PLADs, such as Guangdong and Zhejiang, were more likely to be businessmen instead of farmers and workers than those in less developed PLADs. Farmers, especially those located in border areas, may work in areas where dengue fever is endemic and neglect the protection of mosquito bites during their work abroad, thus risking dengue fever. Personnel engaged in commercial services may be involved in overseas commercial trade and cooperation activities, especially in the context of the rapid development of economic globalization; and there will be increasingly more transnational economic and trade exchanges. If businessmen are not aware of dengue fever protection, then the risk of dengue fever will be very high in countries and regions where dengue fever is endemic. Housework or unemployed people do not have stable jobs but have plenty of free time, so they may have more time to travel to other countries around China. Furthermore, this group of people may follow their family members working abroad to temporarily live abroad. Workers may be dispatched abroad by their company or have stable jobs abroad and return home during vacation or after work. Students may follow their parents to go abroad or study abroad. Public officials may travel abroad for business or travel abroad during vacations. These people may not possess sufficient knowledge about dengue fever prevention and control, and they may not have taken preventive measures to prevent mosquito bites overseas and thus become infected with dengue fever. Therefore, publicity and education on dengue prevention and control knowledge for entry and exit personnel, especially for people in countries or regions where dengue fever is prevalent, should be strengthened. It is also important to familiarize residents in risk areas with the common symptoms of dengue fever and to seek timely medical treatment.

This study combined spatial autocorrelation analysis and temporal-spatial scanning to explore the spatial clustering of overseas imported dengue fever cases in 13 PLADs in China. The results exhibited a trend of more cases in the south and less in the north, more cases in border and coastal areas, and fewer cases inland. The global autocorrelation revealed a spatially positive correlation, suggesting an uneven distribution of these imported cases and the presence of case clusters. This phenomenon may be related to the population flow, international business and employment, studying abroad, and some environmental factors, such as geographic location. The local autocorrelation identified the areas with outliers in southern Yunnan, northern Guangdong and some provincial capitals. The number of outliers in 2013–2019 decreased compared with that in 2005–2012. The clustering areas were mostly distributed in the border areas of Yunnan, Hubei, Henan and Shandong. The border areas of Yunnan and some coastal areas are high-value clusters while the other areas are low-value clusters. The number of high-high clusters and low-low cluster areas in 2013–2019 increased compared with that in 2005–2012.

Hot spots were identified in the southern and southwestern regions of Yunnan Province from 2005 to 2019. These regions are high-risk areas for imported dengue fever. Stringent measures should be implemented to prevent and control imported cases and public health education regarding dengue fever should be strengthened in these regions. From 2005 to 2012, most of the northern part of Guangdong and some coastal areas of Fujian were identified as hotspots, but by 2013–2019, most of the northern part of Guangdong and some coastal areas of Fujian were no longer hotspots. The temporal-spatial scan results obtained by SaTScan are different from Getis-Ord Gi* analysis, which may be due to their different principles [46]. The temporal-spatial scan also showed that the cluster areas of imported cases in 2013–2019 expanded northward compared with those in 2005–2012. Although we do not know whether the trend will continue in the future, attention should also be paid to the northern regions amid global warming and climate change. Global autocorrelation grasps the spatial relationship between imported cases as a whole, and local autocorrelation explores the relationship between imported cases at each city level. SaTScan can scan to the specific range of imported cases through a circular scanning window. The three methods of global autocorrelation, local autocorrelation and SaTScan gradually progress to make the results more comprehensive.

Imported dengue fever cases are the most important driver of dengue outbreaks in China. For better prevention and control of dengue fever, knowledge of dengue fever should be publicized for entry and exit personnel to increase their awareness of the disease. Greater health monitoring and surveillance should be implemented, particularly for people from dengue fever epidemic areas or countries. Regular training for medical personnel in private clinics, township health centers, community hospitals, and large central hospitals to improve their knowledge on dengue fever and their abilities to identify dengue fever is also necessary. This will help to achieve early detection, early diagnosis and early treatment of suspected dengue fever. It is pivotal to collaborate with other countries, particularly those experiencing dengue fever epidemics, by providing technical assistance, training relevant professional personnel, exchanging knowledge and sharing experience in dengue fever control. These efforts will eventually prevent the further spread of dengue fever and minimize the disease burdens resulting from dengue fever.

The study described and analyzed the overseas imported dengue fever cases recorded in 13 PLADs. It is too difficult to count the possible unreported asymptomatic dengue patients, which is a limitation of this study. In addition, the study only described and analyzed the epidemiological characteristics and temporal-spatial distribution of overseas imported dengue fever cases from 13 PLADs with local dengue outbreaks in the mainland of China, but the reasons behind the above epidemiological characteristics and temporal-spatial distribution were not further analyzed. Future research should focus on collecting data on socioeconomic factors such as population entry and exit through different modes of transportation, transnational tourism, economy and trade and meteorological data. Combining these data with the data of overseas imported cases and exploring the factors affecting the epidemic characteristics and temporal-spatial distribution of overseas imported cases will enable more effective prevention and control of overseas imported cases.

## Conclusions

In this study, we characterized the imported dengue fever cases from 2005 to 2019 in 13 PLADs in China and identified the hot spots of these imported cases, which include the border areas of Yunnan, coastal areas of Fujian and Guangdong, and the area of imported cases tended to expand to the north. These findings provide valuable information for developing strategies and policies to prevent and control dengue fever.

## Supplementary Information

**Additional file 1.** Schematic diagram of the study areas.

**Additional file 2.** Classification of occupations.

## Data Availability

The datasets used and analyzed during the current study are available from the corresponding author on reasonable request.
