# Supplementary material for: Epidemiological characteristics and temporal-spatial analysis of overseas imported dengue fever cases in outbreak provinces of China, 2005–2019
Source: Infect Dis Poverty. 2022 Jan 24;11:12. doi: 10.1186/s40249-022-00937-5 (PMC8785556; doi:10.1186/s40249-022-00937-5)
Supplement: Supplementary file 1 — Additional file 1. Schematic diagram of the study areas. [file 40249_2022_937_MOESM1_ESM.pdf]

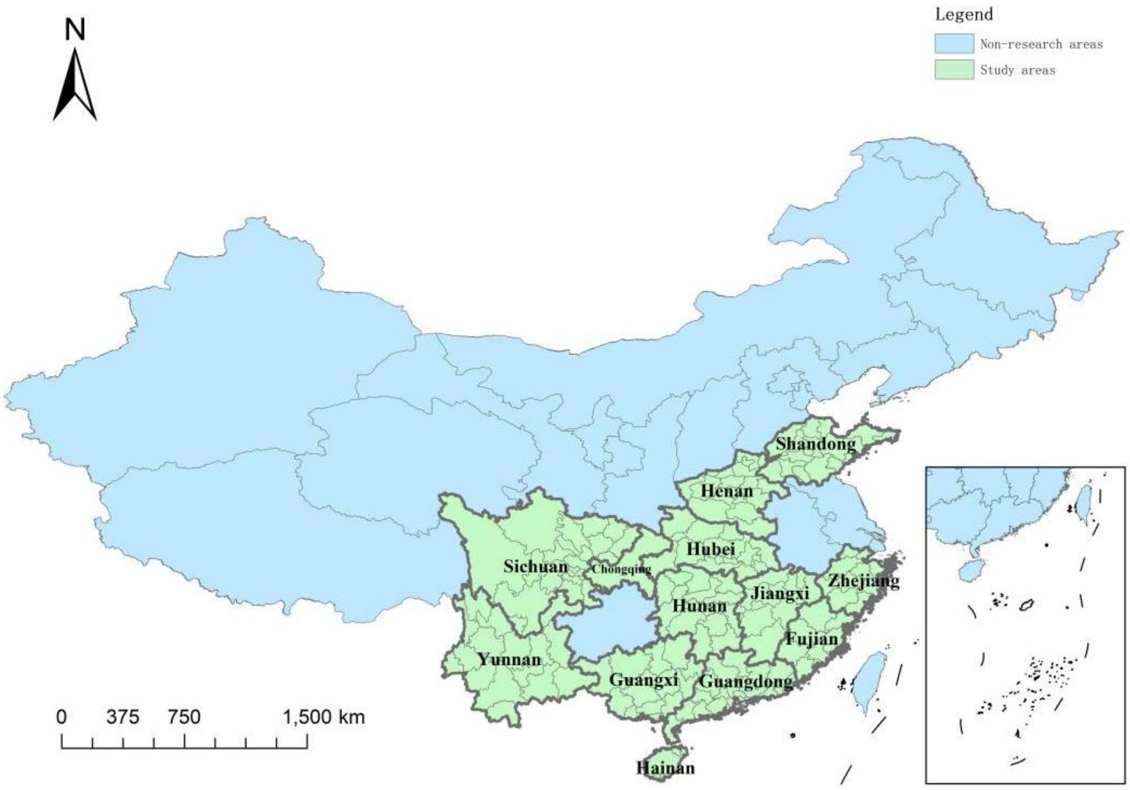

Schematic diagram of the study areas indicating where dengue fever local outbreaks have been recorded in mainland China.
