# Supplementary material for: Epidemiological characteristics and temporal-spatial analysis of overseas imported dengue fever cases in outbreak provinces of China, 2005–2019
Source: Infect Dis Poverty. 2022 Jan 24;11:12. doi: 10.1186/s40249-022-00937-5 (PMC8785556; doi:10.1186/s40249-022-00937-5)
Supplement: Supplementary file 2 — Additional file 2. Classification of occupations. [file 40249_2022_937_MOESM2_ESM.docx]

Classification of occupations

The infectious disease report information management system divides patients’ occupations into the catering industry, cadre, worker, housework or unemployed, teacher, retiree, migrant worker, farmer, scattered children, business service, student, medical staff, children in kindergartens, fisherman, unknown and other categories. We merged similar occupations into one category. Migrant workers and fisherman were merged into farmers; the catering industry was merged into business services; scattered children and children in kindergartens were merged into students; and cadres, retirees, teachers, and medical personnel were merged into public officials. Finally, they were divided into six categories: farmers, business services, housework or unemployed, workers, students and public officials.
